# Acute Effects of Whole-Body Electromyostimulation on Energy Expenditure at Resting and during Uphill Walking in Healthy Young Men

**DOI:** 10.3390/metabo12090781

**Published:** 2022-08-24

**Authors:** Unai A. Perez-De-Arrilucea-Le-Floc’h, Manuel Dote-Montero, Abraham Carle-Calo, Guillermo Sánchez-Delgado, Jonatan R. Ruiz, Francisco J. Amaro-Gahete

**Affiliations:** 1 PROFITH (PROmoting FITness and Health through Physical Activity) Research Group, Sport and Health University Research Institute (iMUDS), Department of Physical Education and Sport, Faculty of Sport Sciences, University of Granada, 18007 Granada, Spain; 2 EFFECTS-262 Research Group, Department of Physiology, Faculty of Medicine, University of Granada, Avenida de la Investigación N° 11, 18007 Granada, Spain; 3 Pennington Biomedical Research Center, Baton Rouge, LA 70808, USA; 4 Department of Medicine, Division of Endocrinology, Université de Sherbrooke, Sherbrooke, QC J1H 5N4, Canada; 5 Instituto de Investigación Biosanitaria, ibs.Granada, 18016 Granada, Spain

**Keywords:** energy metabolism, respiratory exchange ratio, impulse frequencies, whole-body electromyostimulation

## Abstract

The effects of the different electrical frequencies of whole-body electrical stimulation (WB-EMS) on energy expenditure (EE) and the respiratory exchange ratio (RER) remain poorly understood. This study aimed to determine the effects of different WB-EMS electrical frequencies on EE and the RER during supine resting and uphill walking. A total of 10 healthy and recreationally active men (21.6 ± 3.3 years old) participated in the present study. Participants completed two testing sessions in a randomized order. In each session, a variety of impulse frequencies (1 hertz (Hz), 2 Hz, 4 Hz, 6 Hz, 8 Hz, and 10 Hz) were applied in a randomized order, allowing a 10 min passive recovery between them. Oxygen consumption and carbon dioxide production were measured to calculate EE and the RER. All frequencies increased EE at rest (all *p* ≤ 0.001), with 4 Hz being the frequency producing the highest increase (Δ = 8.89 ± 1.49 kcal/min), as did 6 Hz (Δ = 8.05 ± 1.52 kcal/min) and 8 Hz (Δ = 7.04 ± 2.16 kcal/min). An increment in the RER at rest was observed with 4 Hz, 6 Hz, 8 Hz and 10 Hz (all *p* ≤ 0.016), but not with 1 Hz and 2 Hz (*p* ≥ 0.923). During uphill walking, the frequency that elicited the highest increase in EE was 6 Hz (Δ = 4.87 ± 0.84 kcal/min) compared to the unstimulated condition. None of the impulse frequencies altered the RER during uphill walking. WB-EMS increases EE in healthy young men both during resting and uphill walking.

## 1. Introduction

Obesity is a public health problem worldwide since it is a risk factor for cardiovascular diseases and mortality [1,2,3,4]. Excess body weight is mostly explained by an imbalance between energy intake and energy expenditure (EE) [5]. Body weight regulation is highly influenced by genetics, physiology, and socioeconomic factors [6,7]. For instance, low EE (adjusted by body composition) is a known risk factor for increasing body weight and developing obesity [8,9,10]. Similarly, adaptive thermogenesis, which is a reduction in EE beyond what can be predicted by changes in body weight and composition, is a barrier for further weight loss and might contribute to weight regain [11]. In contrast, it seems that high EE achieved through physical activity can help to sustain weight loss [12]. Therefore, interventions capable of increasing EE are of interest for managing body weight and preventing/treating obesity [13,14].

It is well known that physical exercise is an effective strategy to (i) increase EE, (ii) improve body composition [15,16,17,18,19] and physical fitness [20,21,22], and (iii) reduce cardiometabolic risk factors [23,24,25]. Indeed, the World Health Organization has recently changed its physical activity guidelines recommending the combination of endurance (i.e., 150–300 min (min) of moderate and vigorous intensity) and resistance exercise (i.e., >2 sessions/week) [26]. Unfortunately, most people remain inactive [27], commonly eluding to time constraints, the risk of injuries, and a lack of enthusiasm as barriers to sustaining exercise [28,29,30]. Exercise modalities that consume less time could be potential solutions to increase adherence to physical activity. In this context, whole-body electromyostimulation (WB-EMS) training, which produces involuntary contractions in up to 14–18 regions or 8–12 different muscle groups, is becoming increasingly popular worldwide as a potential and attractive alternative to traditional training methods to increase EE [31,32] and to therefore improve body composition and cardiometabolic health [33,34,35].

Only a few studies have investigated the effects of electromyostimulation (EMS) on EE and the respiratory exchange ratio (RER) at rest. For instance, Grosset et al. [36] reported that 1 h of lower limb EMS applying 5 hertz (Hz) at rest increased EE (Δ ≈ 428%) and the RER (Δ ≈ 16%). Minogue et al. [37] also showed that 4 min of local 12 Hz EMS in the quadriceps increases EE (Δ ≈ 596%). However, both studies used only one frequency and local EMS. It remains unknown whether different electrical frequencies applied with WB-EMS at rest produce different EE and RER responses. Regarding the effect of EMS during exercise, Kemmler et al. [32] showed that the application of high frequency (i.e., 80–85 Hz) WB-EMS in a 16-min training session consisting of slight weight-bearing movements increased EE (Δ ≈ 17%) as compared to the same exercise without WB-EMS. Interestingly, Kemmler et al. [32] applied high frequencies (i.e., 80–85 Hz) at the subject’s maximum tolerance levels. Similarly, Verch et al. [38] applied 85 Hz WB-EMS during 10 min of walking and Nordic walking finding an ≈10% increase in EE.

Electrical frequency appears to be inversely proportional to electrical intensity, which is commonly applied at a participant’s maximal tolerance and highly influences the effects of WB-EMS [39]. Hence, we hypothesized that applying lower frequencies will allow higher intensities and higher energy expenditure. The optimal electrical frequency to elicit the highest EE at supine resting and during uphill walking is currently unknown. Therefore, the present study aimed to determine the effects of different WB-EMS electrical frequencies in EE and the RER at supine resting and during uphill walking.

## 2. Materials and Methods

### 2.1. Participants

Ten healthy and recreationally active males (18–25 years old) participated in the present study. The inclusion criteria were: (i) no previous experience with WB-EMS training, (ii) having a stable body weight (variation of <5 kg in body weight over the previous 3 months), (iii) to show a normal weight status (body mass index (BMI) between 18.5–25.0 kg/m^2^), (iv) not taking medications, (v) any chronic metabolic disease or cancer, and (vi) not suffering from any health problem that might be aggravated by exercise or WB-EMS, such as total endoprosthesis, epilepsy, and abdomen/groin hernia. The participants signed a written informed consent before participation and were fully aware of the nature of the study. The study was approved by the Human Research Ethics Committee of the University of Granada (N° 1092/CEIH/2020), registered as a clinical trial (NCT05218512), and was conducted following the latest revision of the Declaration of Helsinki (i.e., 2013).

### 2.2. Design

**Figure 1:**
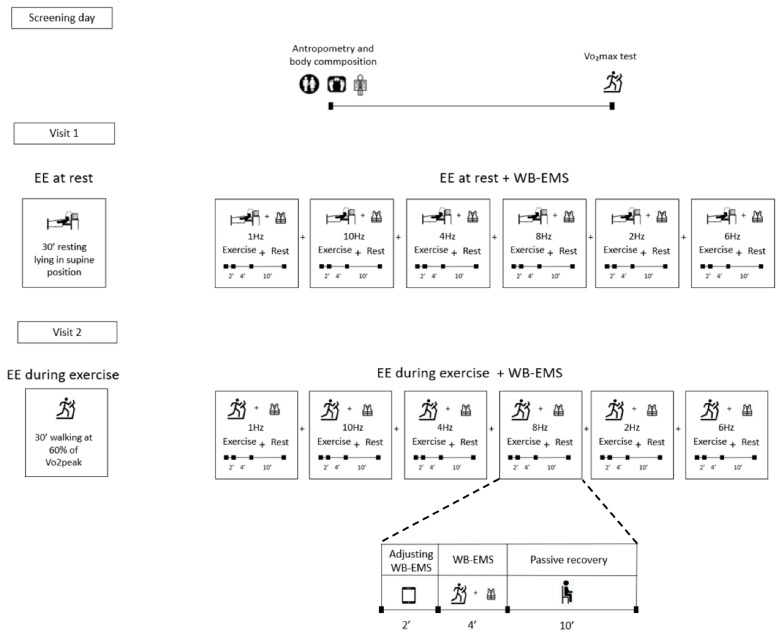
Study design. The different impulse frequencies were applied in a random order. Abbreviations: VO_2_max, maximal oxygen uptake, EE, energy expenditure, WB-EMS, whole-body electromyostimulation.

A within-subject repeated measures design was used to compare the effects of different WB-EMS frequencies on EE and RER at rest and during uphill walking (Figure 1). A wide range of impulse frequencies (i.e., 1 Hz, 2 Hz, 4 Hz, 6 Hz, 8 Hz and 10 Hz) was applied in a randomized order, interposed by subsequent 10-min recovery periods. After an initial screening session, participants completed two testing sessions, seven days apart. The study was conducted between October and November 2018 in the Sport and Health University Research Institute (iMUDS), Granada, Spain. On day 1 (screening), participants went through anthropometric and body composition measurements followed by a graded exercise test to determine the maximal oxygen uptake (VO_2_max) or peak oxygen uptake (VO_2_peak). After seven days, on day 2 (first experimental session), EE and RER were assessed at resting and unstimulated conditions during 30 min followed by the application of 6 different impulse frequencies, in random order, during 6 min each one and with a passive recovery of 10 min between them. During the first 2 min of the 6 min application period, we adjusted the intensity (mA) at the participant’s maximum tolerance, using the last 4 min of the bout to measure the effects of WB-EMS. On day 3 (second experimental session), EE and RER were assessed during 30 min of uphill walking on a treadmill at a speed and grade eliciting an intensity of 60% of the VO_2_max or VO_2_peak (without WB-EMS). The same 6 impulse frequencies were tested subsequently while the participants walked on the treadmill, with 10-min recovery periods in between. Notably, the passive recovery was different in both experimental sessions. During the first experimental session, they waited 10 min lying on the stretcher without moving, while during the second experimental session, they waited 10 min seated steadily in a chair.

The order of the impulse frequencies was randomly selected and counterbalanced. Participants followed the same order application on both days. Participants were instructed to replicate the same diet the day before each visit, to refrain from moderate (previous 24 h) and vigorous physical activity (previous 48 h), and to abstain from alcohol and caffeine consumption (previous 12 h). All measurements were conducted in a temperature-controlled room (22–24 °C) and performed by the same researchers.

### 2.3. Procedures

#### 2.3.1. Anthropometry and Body Composition

Body weight and height were measured using a Seca scale and stadiometer (Seca 799, GmbH & Co. KG, Hamburg, Germany) with participants being barefoot and wearing light clothing. Body composition was assessed by dual-energy X-ray absorptiometry using a Discovery Wi scanner (Hologic, Inc., Bedford, MA, USA), obtaining fat and lean mass. The fat mass index (FMI) and lean mass index (LMI) were calculated as fat body mass (kg)/height (m^2^) and lean body mass (kg)/height (m^2^), respectively.

#### 2.3.2. VO₂max Test

VO_2_max was assessed during a maximum treadmill (H/P/Cosmos Pulsar treadmill, H/P/Cosmos Sport and Medical GMBH, Germany) exercise test with a progressive incremental protocol that has been extensively used and validated [40,41,42,43]. The participants performed the modified Balke protocol [44] with 3 min stages. First, we estimated the maximum speed that allows comfortable walking (i.e., 4.5 km/h, 5.5 km/h, 6.5 km/h, or 7.5 km/h). Thereafter, they warmed up, starting at 3.5 km/h and increasing 1 km/h every 3 min until the aforementioned speed was reached. Then, the grade was increased 2% every 3 min until volition exhaustion. Oxygen consumption (VO_2_) and carbon dioxide production (VCO_2_) were recorded using a Vyntus CPX Metabolic Cart (Vyaire, Hochberg, Germany). Accuracy and reliability of Vyntus CPX has been previously tested [45]. The study of Alcantara et al. showed no differences in EE at rest reproducibility in 29 young non-ventilated adults (5.0 ± 5.6%) and an error for EE measurements of 13.8 ± 5.0% in controlled pure gas infusions and methanol burns. Participants were encouraged to invest maximum effort and provided measures of their rating of perceived exercise during the last 15 s (sec) of each stage, using the 6–20 Borg scale [46]. The achievement of VO_2_max was established as [47]: (i) showing a VO_2_ change < 100 mL/min in the last 30 s of the final stage, (ii) attaining an RER ≥ 1.1, and (iii) reaching a heart rate between ±10 beats/min of the theoretical maximal heart rate. The VO_2_peak was considered when these criteria were not met [48] and all participants achieved the previously mentioned criteria.

#### 2.3.3. Energy Expenditure and RER at Rest

EE and RER were measured while resting, laying in a supine position, between 8 A.M. and 11 A.M. after a 12-h overnight fasting. The participants arrived at the research center by public transportation or by any motor vehicle avoiding any physical activity since waking up. We measured EE and RER in control conditions during a 30-min period with the above-mentioned metabolic cart. The participants were instructed not to move throughout the entire test. A silicone face mask with a twin-tube sample line and a digital volume transducer was used for gas data collection. The measurements were subsequently recorded at 10 s intervals for VO_2_ and VCO_2_. The gas analyzer was calibrated before every test using the manufacturer’s automated flow and digital volume transducer calibration (i.e., 15.92% O_2_ and 5.03% CO_2_). The indirect calorimetry’s measurement was performed in agreement with the recommended guidelines [49]. Briefly, the participants were assessed in the same room, with controlled ambient temperature (22–24 °C), and by the same trained researchers. They laid on a reclined stretcher in a supine position for a minimum of 15 min before the EE and RER at rest measurement. Furthermore, the participants were instructed to breathe normally, and not to fidget, talk, or sleep while measurements were being taken.

For control (i.e., unstimulated) measurements, the first 5 min data were discarded, and records with an RER <0.7 or >1.0 were excluded. The coefficients of variance for VO_2_, VCO_2_ ventilation and RER were calculated, and the periods that met the steady-state criteria (i.e., CV < 10% for VO_2_, VCO_2_, and ventilation and CV < 5% for RER) were then selected. Finally, the period with the lowest CV was chosen for further analysis. EE was calculated using the stoichiometry equations of Weir [50] and expressed in kcal/min. Despite participants wearing the face mask during the whole WB-EMS stimulated resting time, only the last 4 min of each stimulation bout were considered in further analyses.

#### 2.3.4. Energy Expenditure and RER during Uphill Walking

EE and RER were measured while uphill walking on a treadmill. The grade of the treadmill was personally adjusted to the one that elicited 60% of the VO₂max/peak during the maximum effort test. The participants arrived in the same conditions as the first experimental session and had their unstimulated walking EE measured during 30 min followed by the application of the same order of impulse frequencies used in the first experimental session. Data analysis was performed following the same steps as during resting conditions.

#### 2.3.5. Whole-Body Electromyostimulation Protocol

**Figure 2:**
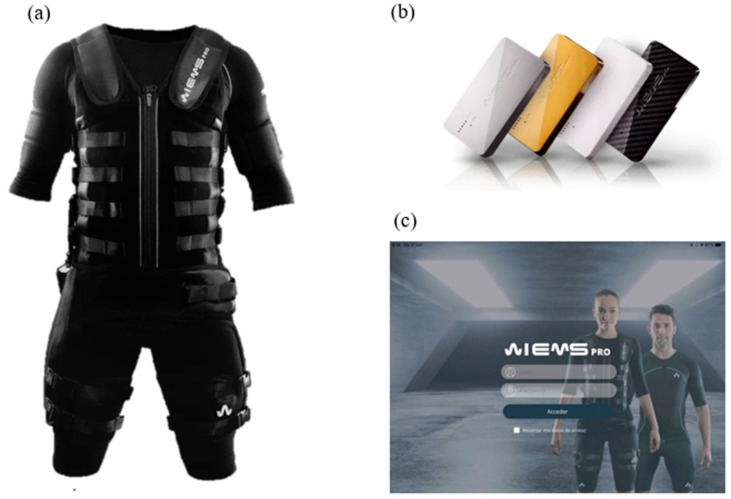
(**a**) Whole-body electromyostimulation suit; (**b**) whole-body electromyostimulation devices that transmit electrical stimuli; (**c**) whole-body electromyostimulation app to configure electrical stimuli.

The WB-EMS protocol was performed using an electromyostimulation device (Wiemspro^®^, Malaga, Spain), which simultaneously stimulates 8 muscle groups (i.e., upper legs, upper arms, upper back, gluteal, abdomen, chest, lower back, and shoulders; total size of electrodes: 2800 cm^2^) (Figure 2).

Since no previous studies have investigated whether the application of different WB-EMS impulse frequencies modifies EE and RER at rest and during uphill walking, we conducted a pilot study selecting a large variety of impulse frequencies, ranging from 1 to 100 Hz. This pilot testing showed that lower frequencies (≤10 Hz) induced higher EE than those observed with high frequencies (>20 Hz). Therefore, we restricted the frequency range applied in the present study from 1 to 10 Hz.

Several electrical parameters were set: (a) the impulse frequency (i.e., the number of electrical pulses per time unit, measured by Hz); (b) impulse intensity (i.e., the quantity of electricity, measured in milliamps—mA); (c) impulse width (i.e., the time of each impulse, measured in microseconds—μsec); (d) duty cycle (i.e., the ratio between time receiving electrical stimuli and the total cycle time (% duty cycle = 100/(total time/on time))). Impulse intensity (mA) was adjusted at the participant’s maximum tolerance during the first 2 min of the 6 min application period [32,51]. Participants were asked to report the intensity of the electric impulse and the perceived intensity by using the reported perceived exertion (RPE) scale [46]. Impulse width was kept fixed following general recommendations from each muscle group [52] (i.e., arms = 200 μsec, cervical = 200 μsec, chest = 200 μsec, dorsal = 250 μsec, abdominal = 300 μsec, glutes = 350 μsec, and thighs = 400 μsec). Finally, the duty cycle was fixed at 99% to find the physiological response to a continued electrical impulse. RPE and Visual Analogue Scale (VAS) were used to register pain perception just after the application of each frequency.

### 2.4. Statistical Analyses

Descriptive variables are reported as mean ± standard deviation. The normality of the distribution of all variables was assessed by the Shapiro–Wilk statistic, visual check of histograms, and Q–Q plots. The data followed a normal distribution and, as a result, a repeated measures analysis of variance (ANOVA) was used to compare the EE and RER elicited by different impulse frequencies (i.e., 1 Hz, 2 Hz, 4 Hz, 6 Hz, 8 Hz, and 10 Hz) at rest and during uphill walking. The Mauchly test indicated that the sphericity assumption (homogeneity) was met for the effects of impulse frequencies on the EE and RER at rest and during uphill walking (*p* > 0.05).

The effect size was measured by partial eta squared (ƞ²), and classified as small, medium, or large (<0.06, 0.06–0.14, and >0.14, respectively), following established guidelines [53]. Post hoc Bonferroni tests with adjustment were performed to examine the difference between impulse frequencies.

Significance was set at *p* ≤ 0.05. All analyses were performed using the Statistical Package for the Social Sciences (SPSS, v. 25.0, IBM SPSS Statistics, IBM Corporation, Armonk, NY, USA). Graphical presentations were prepared using GraphPad Prism 8 software (GraphPad Software, San Diego, CA, USA).

## 3. Results

Descriptive data of the participants are shown in Table 1. There were no WB-EMS-related adverse effects during the study course. Two participants did not perform the exercise part due to medical reasons (they were sick for personal reasons).

### 3.1. Effects of WB-EMS on Energy Expenditure at Rest

**Figure 3:**
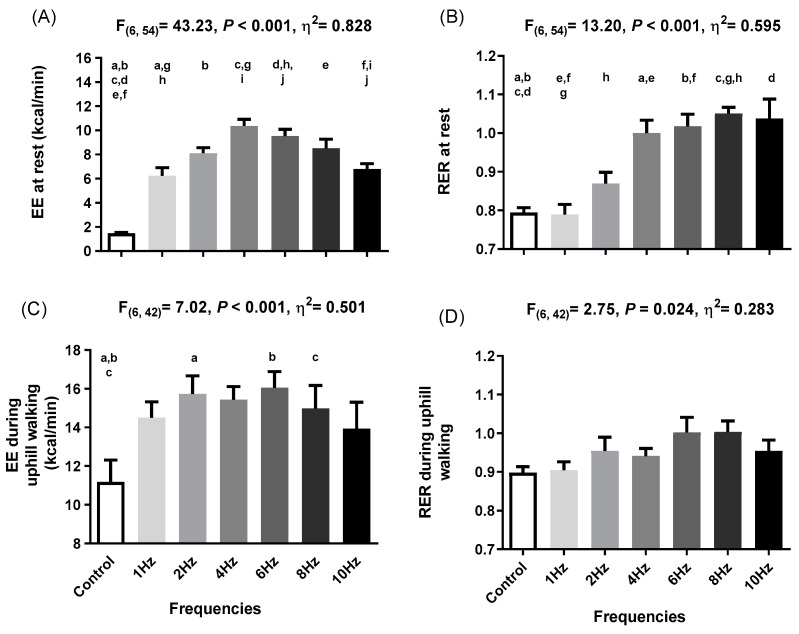
(**A**) Energy expenditure (EE) at rest (n = 10) when applying different frequencies of whole-body electromyostimulation; (**B**) respiratory exchange ratio (RER) at rest (n = 10) when applying different frequencies of whole-body electromyostimulation; (**C**) EE during uphill walking (n = 8) when applying different frequencies of whole-body electromyostimulation; (**D**) RER during uphill walking when applying different frequencies of whole-body electromyostimulation. EE when applying different frequencies of whole-body electromyostimulation. *p*-values from repeated measures analysis of variance (ANOVA). Similar letters represent differences between experimental conditions as determined by post hoc Bonferroni analysis.

There were significant differences in EE at rest across impulse frequencies (F (6,54) = 43.23, *p* < 0.001, ƞ² = 0.828). Post hoc analyses indicated differences in EE between unstimulated and all the stimulated impulse frequencies (all *p* ≤ 0.001; Figure 3a). We noted an increase in EE from 1.47 kcal/min at rest conditions to 6.23 kcal/min (Δ = 322.8%) at 1 Hz, 8.11 kcal/min (Δ = 450.8%) at 2 Hz, 10.36 kcal/min (Δ = 603.6%) at 4 Hz, 9.52 kcal/min (Δ = 546.6%) at 6 Hz, 8.51 kcal/min (Δ = 477.9%) at 8 Hz, and 7.37 kcal/min (Δ = 361.3%) at 10 Hz. There were also significant differences in the RER at rest across impulse frequencies (F (6,54) = 13.20, *p* < 0.001, ƞ² = 0.595). Post hoc analyses indicated differences between the unstimulated period and 4 Hz (Δ = +0.21; Δ = 25.9%; *p* =0.008), 6 Hz (Δ = +0.22; Δ = 28.1%; *p* = 0.004), 8 Hz (Δ = +0.26; Δ = 32.3%; *p* < 0.001), and 10 Hz (Δ = +0.24; Δ = 30.6%; *p* = 0.016) (Figure 3b). We conducted an individual analysis of the EE and RER at rest to observe the differences between participants (Figure S1a,b).

There were significant differences between frequencies in participants’ pain perception (F (5,29) = 9.48, *p* < 0.001, ƞ² = 0.613) at rest. Post hoc analysis showed that there were notable disparities in perceived pain between control conditions and 6 Hz (*p* = 0.001) and 10 Hz (*p* = 0.013) in the majority of the body muscles (Figure S2). There were no significant differences between the intensities applied on each frequency (all *p* > 0.071) except for 2 Hz and 8 Hz in whole body intensity (*p* = 0.011), upper back (*p* = 0.026), lower back (*p* = 0.009), gluteal (*p* = 0.044), and hamstrings (*p* = 0.044) at rest (Figure S3).

### 3.2. Effects of WB-EMS on Energy Expenditure during Uphill Walking

There were significant differences across impulse frequencies in EE during uphill walking (F (6,42) = 7.02, *p* < 0.001, ƞ² = 0.501). Post hoc analyses indicated differences in EE between the unstimulated condition and 2 Hz, 6 Hz, and 8 Hz (all *p* ≤ 0.027) but not for 1 Hz, 4 Hz, and 10 Hz (all *p* ≥ 0.063) (Figure 3c). We observed an increase in EE from 11.18 kcal/min during unstimulated uphill walking to 15.73 kcal/min (Δ = 38.1%) at 2 Hz, 16.05 kcal/min (Δ = 43.56%) at 6 Hz, and 14.97 kcal/min (Δ = 33.94%) at 8 Hz. There were also significant differences across impulse frequencies in RER during uphill walking (F (6,42) = 2.75, *p* = 0.024, ƞ² = 0.283). However, post hoc analyses indicated no significant differences in RER between the control conditions and any of the frequencies applied (all *p* ≥ 0.247; Figure 3d). Specific EE and RER data of each participant during uphill walking are shown in Figure S1c,d.

There were significant differences across impulse frequencies in pain perception during uphill walking (F (6,36) = 10.28, *p* < 0.001, ƞ² = 0.632). Post hoc analysis only showed a higher pain perception at 4 Hz in low back (*p* = 0.041), hamstrings (*p* = 0.007), and gluteal (*p* = 0.046) compared to control conditions (Figure S4). There were no significant differences between the intensities applied in the majority of the body muscles during uphill exercise except for gluteal at 1 Hz (*p* = 0.046), 6 Hz (*p* = 0.046), 8 Hz (*p* = 0.007), and 10 Hz (*p* = 0.008) (Figure S5) but not in other muscle groups.

## 4. Discussion

To the best of our knowledge, this is the first study aiming to elucidate which is the WB-EMS impulse frequency that elicits the highest increase in EE at rest and during uphill walking. We observed that 4 Hz induces the highest EE at rest (Δ = 8.89 ± 1.49 kcal/min; Δ = 603.60%), whereas 6 Hz seems to produce the most extensive EE during uphill walking (Δ = 4.87 ± 0.84 kcal/min; Δ = 43.56%). There were also other frequencies that elicit significant increases in EE at rest (e.g., 6 Hz, Δ = 8.05 ± 1.52 kcal/min, Δ = 603.60% and 8 Hz, Δ = 7.03 ± 2.16 kcal/min, Δ = 477.87%) compared to unstimulated conditions and during uphill walking (e.g., 2 Hz, Δ = 4.55 ± 0.54 kcal/min, Δ = 40.70% and 8 Hz, Δ = 3.79 ± 0.18 kcal/min, Δ = 33.94%). A significant increase in the RER at rest was induced by 4 Hz, 6 Hz, 8 Hz, and 10 Hz, but not by low frequencies such as 1 Hz and 2 Hz. Lastly, there were no significant effects of WB-EMS on the RER during exercise. These results suggest that a single bout of WB-EMS at 4 Hz and 6 Hz (adjusting impulse intensity to the participant’s maximum tolerance) induces the highest increase in EE at rest and during uphill walking, respectively. Although 4 Hz seems to be the frequency that elicits the highest increase in EE, there were no significant differences between 4 Hz, 6 Hz, and 8 Hz at rest. Similarly, during uphill walking, we did not find significant differences between 6 Hz, 2 Hz, and 8 Hz. Intriguingly, although 1 Hz and 2 Hz do not change the RER values, they increase EE at rest suggesting a potential impact on substrate utilization.

There are previous studies investigating the impact of local EMS on EE at rest. Grosset et al. [36] compared the EE induced by involuntary skeletal muscle contractions and reported that 1 h of lower limb EMS in adults with obesity (i.e., isometric knee extension contraction at 5 Hz in a lying position without a determined duty cycle and at the maximum tolerable intensity) increased EE rates (Δ = +240.8 kcal/h; ≈428%) as compared to resting conditions. We observed a similar effect in EE, although we found a higher increment (Δ = 603.60%), which is expected since we used WB-EMS instead of local EMS. Minogue et al. [37] tested the effect of local EMS (i.e., symmetric biphasic pulse, intensity of 200 mA, phase duration of 600 μsec, and interphase interval of 100 μsec) in the quadriceps muscles on EE applying different impulse frequencies during 4 min (i.e., 1 Hz, 2 Hz, 4 Hz, 5 Hz, 6 Hz, 8 Hz, 10 Hz and 12 Hz) while sitting at rest. They observed the highest EE at 12 Hz (≈2.8 kcal/min; Δ ≈ 596%), while the EE was increased (≈1.6 kcal/min; Δ ≈ 294%) when selecting 4 Hz. This increase is lower than the one observed in our study for 4 Hz (Δ = 8.89 ± 1.49 kcal/min; Δ = 603.60%). This discrepancy is likely explained because Minogue et al. [37] used local EMS in quadriceps muscles with participants seated, while we applied a WB-EMS protocol while participants were lying. Another reason could be that they used different electrical parameters such as duty cycle (99% vs. 50%) among others.

Regarding WB-EMS stimulation during exercise, Kemmler et al. [32] showed an increase of Δ = +1 kcal/min (≈17%) on EE, while we reported Δ = 4.87 ± 0.84 kcal/min (43.56%). These differences could be due to the exercise performed and WB-EMS settings used. In the study by Kemmler et al. [32], participants performed light weight-bearing movements with high fixed frequencies (i.e., 80–85 Hz), while we performed uphill walking with low frequencies (i.e., 1–10 Hz). We cannot compare the electrical intensity since they did not report the one they applied. On the other hand, Verch et al. [38] aimed to determine the differences in VO_2_ comparing Nordic walking and walking with and without WB-EMS. They continuously applied WB-EMS during 10 min with the following pattern: 9 s at 85 Hz and 1 s at 7 Hz, with a pulse width 350 μsec in every muscle stimulated at the individual tolerated maximum intensity [38]. Interestingly, they found a ≈ 10% increase in VO_2_ elicited by WB-EMS [38]. The comparison between Verch et al.’s [38] findings and our study’s results is hard since electrical parameters and the time of application are not equivalent. However, we observed a much bigger increase in EE than Verch et al. [38].

The RER indicates the prevalence of one substrate utilization (i.e., fat vs. carbohydrate utilization), provided that some assumptions are met [54]. We observed a lower RER when applying 1 Hz and 2 Hz at rest suggesting higher fat utilization compared to the rest of the frequencies. Importantly, these frequencies increased EE without modifying the RER, which suggest that low frequency WB-EMS preferentially induces fat oxidation, a fact that could be extremely interesting since there is enough evidence to think that low fat utilization is a risk factor for developing obesity and weight gain [55]. Higher frequencies are usually associated with a higher activation of fast-twitch type IIX and IIA muscle fibers, which consume glucose almost exclusively and in turn would explain the higher RER [56]. In this sense, Hamada et al. [57] compared the RER after the application of EMS in different conditions: (i) involuntary lower limb muscle contractions at 20 Hz with duty cycle of 1-s stimulation/1-s pause for 20 min vs. (ii) voluntary skeletal muscle contractions consisting of cycling at the same intensity obtained with EMS during 20 min [57]. They found a higher increase in the RER during the application of local EMS at 20 Hz than during the voluntary skeletal muscle contraction [57]. Although the increase in the RER observed by Hamada et al. [57] was similar to the one observed in our study, this comparison should be performed cautiously since the electrical parameters and time of application were quite different. On the other hand, Minogue et al. [37] found the highest increase in the RER at 12 Hz (Δ ≈ +0.16; Δ ≈ 18%), while we obtained the highest increase in the RER at 8 Hz (Δ = +0.26; Δ = 32.3%). Grosset et al. [36] observed an increment in the RER (Δ = 16% ± 4%) in response to a 1-h bout of maximally tolerated low-frequency electrical muscle stimulation at 5 Hz (lying position) in people with obesity. We observed similar RER values using 4 Hz, 6 Hz, 8 Hz, and 10 Hz (Δ ≈ 29% ± 3%), but not with 1 Hz and 2 Hz (Δ ≈ 4% ± 7%), during just 4 min.

Electrical frequencies and intensities are inversely proportional and the relationship between both parameters could explain the differences observed in EE. Intensity has been positioned as a crucial parameter to modulate EE. For instance, Hsu et al. [58] demonstrated that higher impulse intensities induced greater EE rates during the application of local EMS. The effects of WB-EMS are dependent on impulse intensities, and since the above-mentioned studies did not report the intensities applied, we cannot compare them. This electrical parameter could be the reason why we observed different responses in EE and the RER compared to other studies. Based on our results there is a tendency to reduce the intensity while the frequency increases; however, we did not find significant differences in intensity between frequencies in both conditions (i.e., resting and during uphill walking). Moreover, we only found differences in pain perception between 6 Hz and 10 Hz at rest, whereas no differences were noted during uphill walking. Thus, we can assume that the results are neither influenced by intensity nor pain perception. However, when participants remained at rest, the whole-body intensity was higher than when they were walking (i.e., 104 ± 19 mA vs. 62 ± 16 mA; *p* < 0.001) due to the incompatibility of walking with high intensities. This fact would explain why the RER values rise to higher levels at rest than during uphill walking. Concretely, we found that the RER values at 10 Hz at rest were 1.04 ± 0.16, while during uphill walking the were 0.95 ± 0.08 at the same frequency.

We showed that the application of low frequencies of WB-EMS at rest produces an EE level similar to the one observed during uphill walking (i.e., at 60% of the VO₂max). This could be of interest for individuals with severe obesity problems since long aerobic training sessions at moderate intensity could lead to joint and biomechanical imbalance increasing injury risk [59], as well as for people with reduced mobility. It is believed that high EE achieved through physical activity and/or exercise can help to sustain weight loss [12]. Therefore, interventions capable of increasing EE might result in a better management of body weight and obesity [14]. Moreover, since it has been suggested that most exercise interventions are not usually attractive for these patients, in part attributed to the uncomfortable feelings experienced during the exercise session [60,61], WB-EMS training sessions could be an attractive alternative to enhance the motivation of this cohort. However, we cannot underestimate that although our participants did not report extreme pain, they anecdotally reported discomfort while walking with the WB-EMS at maximum intensity, which could also result in poor adherence.

The present study had several limitations. Although we found significant differences between frequencies, the limited sample size should be considered. Moreover, we only measured the effects of each frequency during 4 min, not enough time to talk about substrate oxidation despite being measured (Figure S6). This is an acute study and, for this reason, the data cannot be extrapolated to possible chronic adaptations such as a decrease in fat mass. Furthermore, WB-EMS equipment still has a high price, which means that this technology is not accessible to everyone. Our study only included young male adults with no previous experience with WB-EMS and our results cannot be extended to other populations. Future studies are needed to clarify the effects of these low frequencies on EE and fuel utilization during longer stimulation periods (>4 min), and while performing other types of exercise and/or in other ergometers. It may be beneficial to investigate these effects in other population such as older individuals and patients with obesity.

## 5. Conclusions

The present study shows that relatively low frequencies of WB-EMS are an effective tool to increase EE at rest and during uphill walking in young healthy adults. Specifically, although 4 Hz is the frequency that elicits the major increment in EE at rest, other frequencies such as 2 Hz, 6 Hz and 8 Hz also did this compared to unstimulated conditions. In addition, the frequency that produces the highest increment in EE during uphill walking is 6 Hz, having a similar effect with 2 Hz and 8 Hz, compared to unstimulated conditions. Lastly, low frequencies (1 Hz and 2 Hz) of WB-EMS do not increase the RER values despite an increase in EE at rest suggesting that it could have an impact on fuel utilization rates.

## Figures and Tables

**Table 1:** Descriptive characteristics of the study participants.

	Mean	SD
Age (years)	21.6	(3.3)
**Anthropometry and body composition**		
Weight (kg)	77.0	(13.6)
Height (cm)	178.3	(8.2)
Body mass index (kg/m^2^)	24.2	(3.8)
Lean mass index (kg/m^2^)	17.4	(1.7)
Fat mass index (kg/m^2^)	5.5	(2.4)
Fat mass (%)	22.3	(6.7)
**Cardiorespiratory fitness**		
VO_2_max (mL/min)	3570.0	(555.9)
VO_2_max (mL/kg/min)	46.8	(5.2)
Energy metabolism at rest		
EE at rest (kcal/min)	1.5	(0.2)
EE at rest (kcal/day)	2101.5	344.7
RER	0.794	(0.042)
**Energy metabolism during uphill walking**		
EE (kcal/min)	11.2	(3.2)
RER	0.898	(0.044)

Data are shown as means (standard deviation). EE, energy expenditure; RER, respiratory exchange ratio; VO_2_max, maximum oxygen consumption.

## Data Availability

The data that support the findings of this study are available from the corresponding author, (UAP), upon reasonable request. The data are not publicly available due to privacy restrictions.

## Supplementary Materials

The following supporting information can be downloaded at https://www.mdpi.com/article/10.3390/metabo12090781/s1: Figure S1. Individual values of energy expenditure (EE) and respiratory exchange ratio (RER) at rest (n = 10) and during uphill walking (n = 8) when applying different frequencies of whole-body electromyostimulation. Figure S2. Pain perception in different anatomic locations after applying whole-body electromyostimulation at different frequencies at rest (n = 7). Figure S3. Impulse intensity at rest (n = 6) applying whole-body electromyostimulation with different frequencies. Figure S4. Pain perception in different anatomic locations after applying whole-body electromyostimulation at different frequencies during uphill walking (n = 7). Figure S5. Impulse intensity during uphill walking (n = 7) applying whole-body electromyostimulation with different frequencies. Figure S6. Fat oxidation (FatOx) and carbohydrate oxidation (ChoOx) at rest (n = 10) and during uphill walking (n = 8) when applying different frequencies of whole-body electromyostimulation.
