# Supplementary material for: Acute Effects of Whole-Body Electromyostimulation on Energy Expenditure at Resting and during Uphill Walking in Healthy Young Men
Source: Metabolites. 2022 Aug 24;12(9):781. doi: 10.3390/metabo12090781 (PMC9504893; doi:10.3390/metabo12090781)
Supplement: Supplementary file 1 [file metabolites-12-00781-s001.zip › metabolites-1852603-supplementary.pdf]

Supplementary material

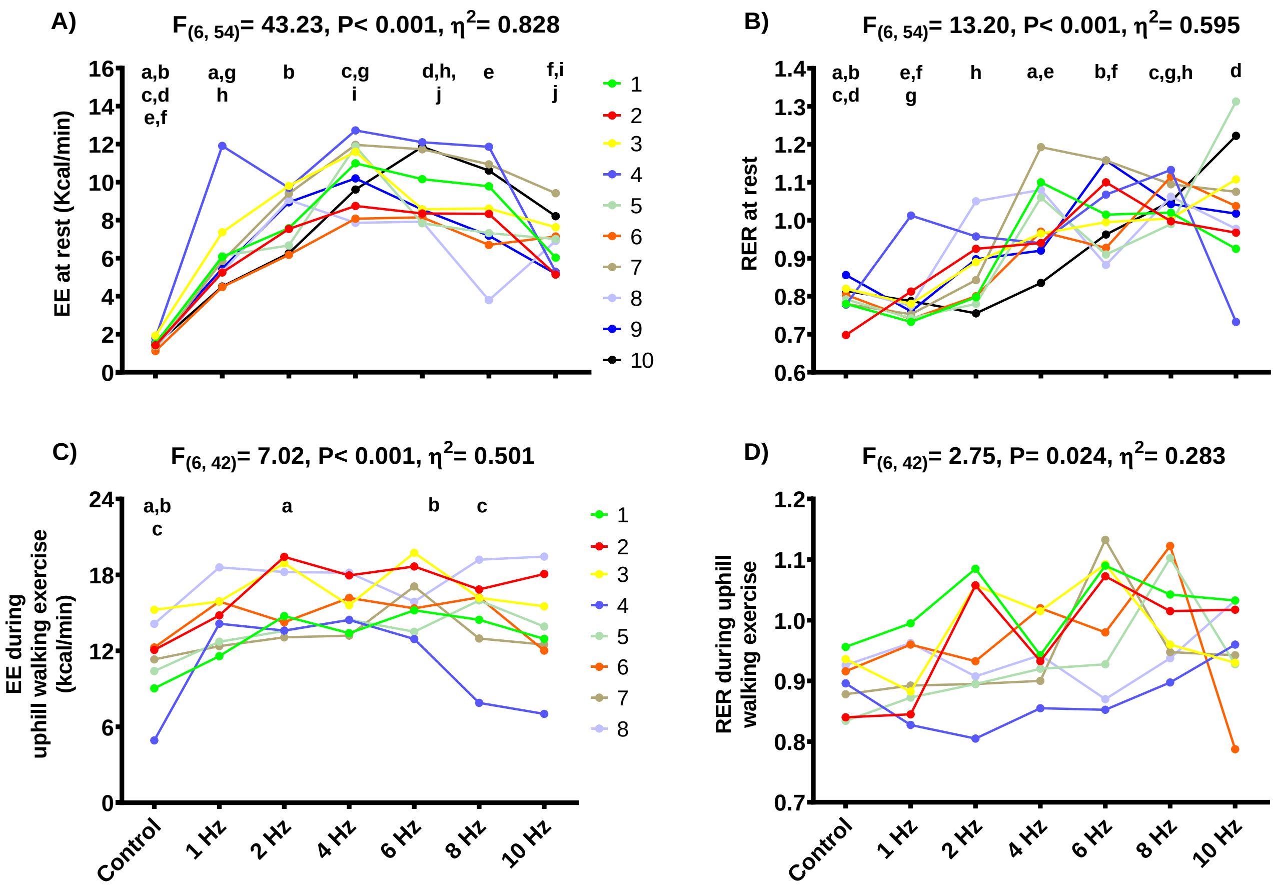

Figure S1. Individual values of energy expenditure (EE) and respiratory exchange ratio (RER) at rest (n=10) and during uphill walking (n=8) when applying different frequencies of whole-body electromyostimulation. *p*-values from repeated measures analysis of variance (ANOVA). Similar letters represent differences between experimental conditions as determined by post-hoc Bonferroni analysis. Crow data are presented as mean of 4 minutes in each frequency

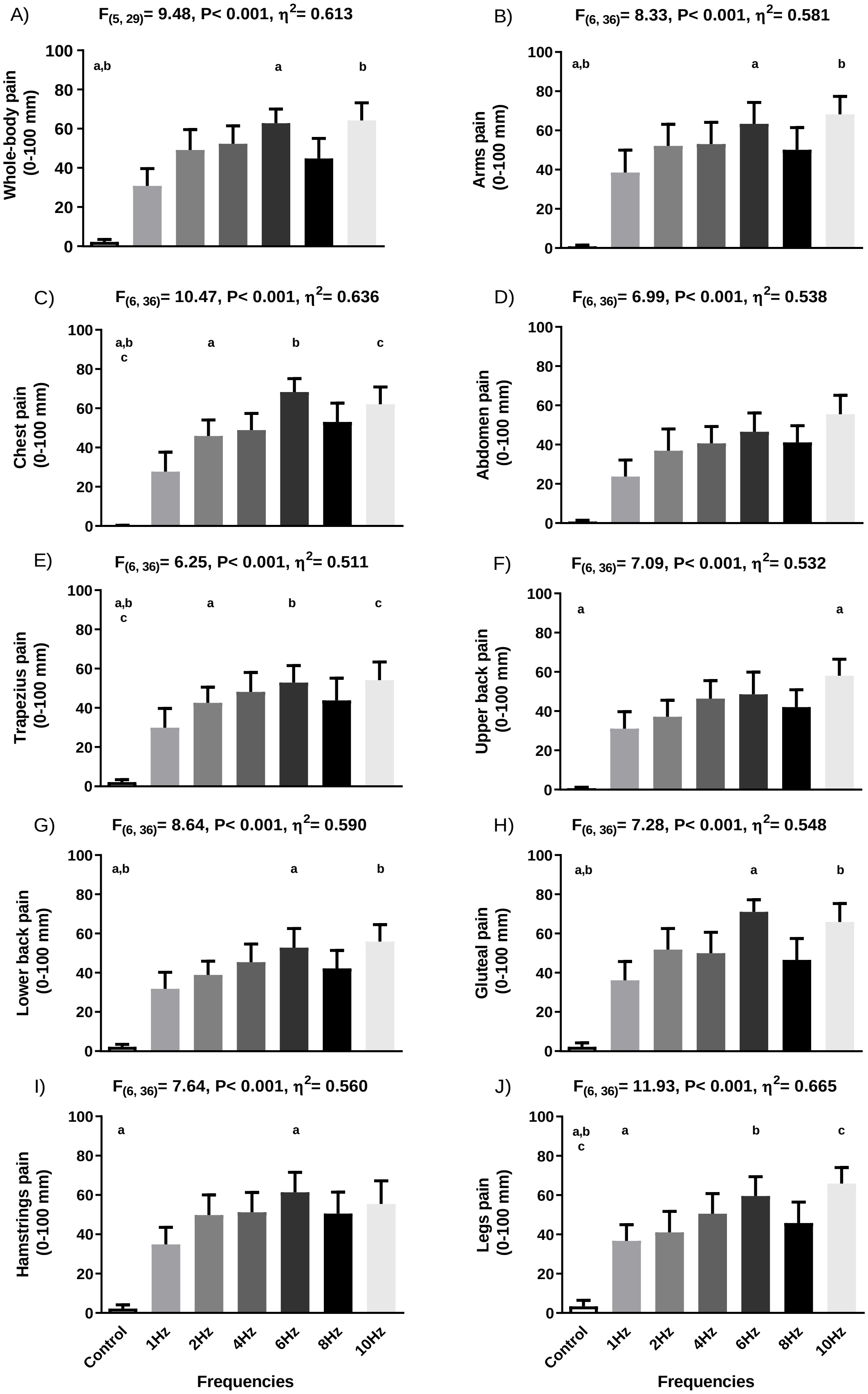

A)
F(5, 29)= 9.48, P< 0.001, η2= 0.613
Whole-body pain (0-100 mm)
a,b
a
b
B)
F(6, 36)= 8.33, P< 0.001, η2= 0.581
Arms pain (0-100 mm)
a,b
a
b
C)
F(6, 36)= 10.47, P< 0.001, η2= 0.636
Chest pain (0-100 mm)
a,b
c
a
b
c
D)
F(6, 36)= 6.99, P< 0.001, η2= 0.538
Abdomen pain (0-100 mm)
E)
F(6, 36)= 6.25, P< 0.001, η2= 0.511
Trapezius pain (0-100 mm)
a,b
c
a
b
c
F)
F(6, 36)= 7.09, P< 0.001, η2= 0.532
Upper back pain (0-100 mm)
a
a
G)
F(6, 36)= 8.64, P< 0.001, η2= 0.590
Lower back pain (0-100 mm)
a,b
a
b
H)
F(6, 36)= 7.28, P< 0.001, η2= 0.548
Gluteal pain (0-100 mm)
a,b
a
b
I)
F(6, 36)= 7.64, P< 0.001, η2= 0.560
Hamstrings pain (0-100 mm)
a
a
J)
F(6, 36)= 11.93, P< 0.001, η2= 0.665
Legs pain (0-100 mm)
a,b
c
a
b
c
0
20
40
60
80
100
Control
1Hz
2Hz
4Hz
6Hz
8Hz
10Hz
Frequencies

Figure S2. Pain perception in different anatomic locations after applying whole-body electromyostimulation at different frequencies at rest (n=7). Visual analogue scales (VAS) ranges from 0 to 100, being 0 "no pain", and 100 "the maximum tolerable pain”. *p*-values from repeated measures analysis of variance (ANOVA). Similar letters represent differences between experimental conditions as determined by post-hoc Bonferroni analysis. Data are presented as mean and standard error of the mean (SEM).

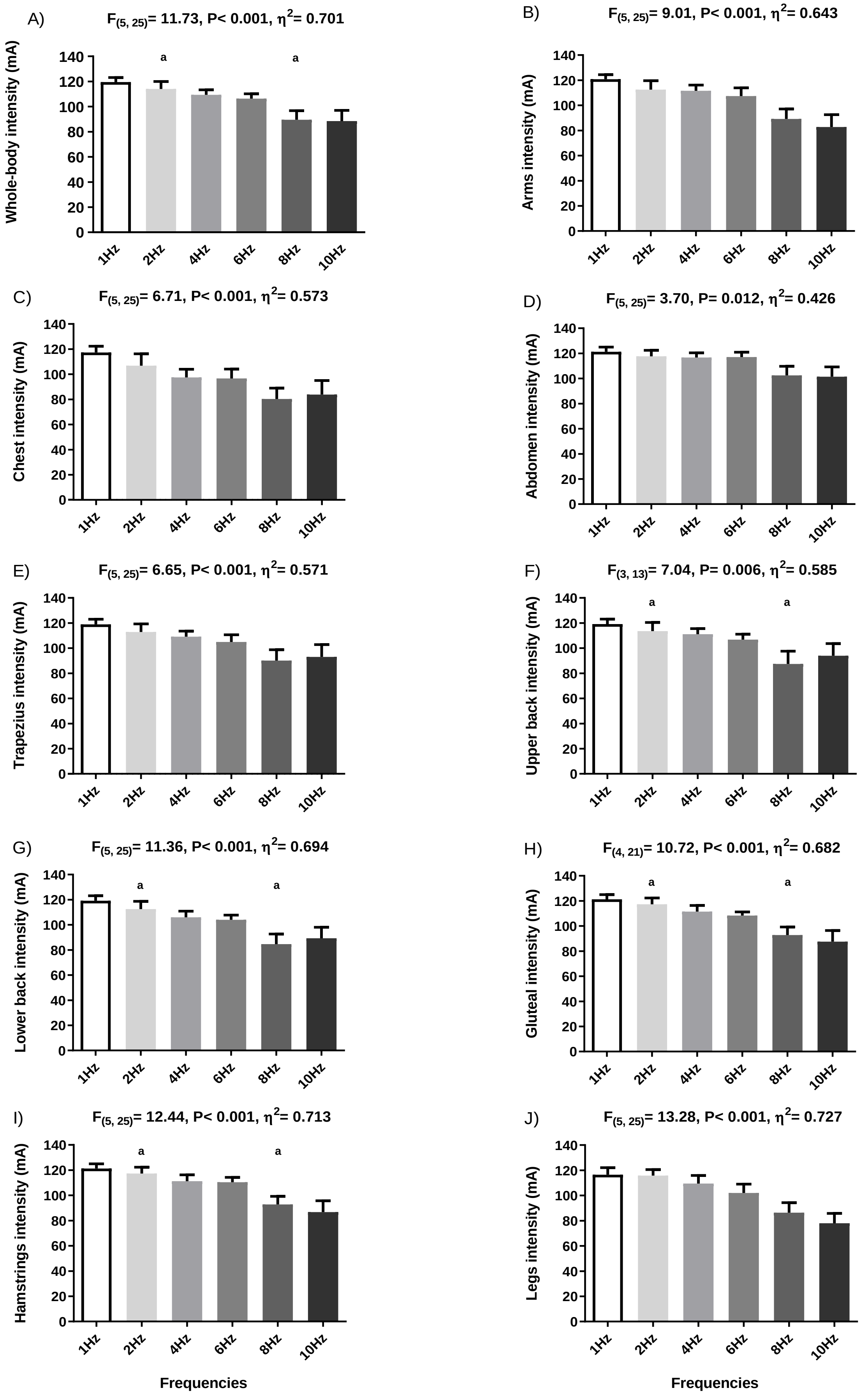
A)
$F_{(5, 25)}$= 11.73, P< 0.001, η^2= 0.701
Whole-body intensity (mA)
B)
$F_{(5, 25)}$= 9.01, P< 0.001, η^2= 0.643
Arms intensity (mA)
C)
$F_{(5, 25)}$= 6.71, P< 0.001, η^2= 0.573
Chest intensity (mA)
D)
$F_{(5, 25)}$= 3.70, P= 0.012, η^2= 0.426
Abdomen intensity (mA)
E)
$F_{(5, 25)}$= 6.65, P< 0.001, η^2= 0.571
Trapezius intensity (mA)
F)
$F_{(3, 13)}$= 7.04, P= 0.006, η^2= 0.585
Upper back intensity (mA)
G)
$F_{(5, 25)}$= 11.36, P< 0.001, η^2= 0.694
Lower back intensity (mA)
H)
$F_{(4, 21)}$= 10.72, P< 0.001, η^2= 0.682
Gluteal intensity (mA)
I)
$F_{(5, 25)}$= 12.44, P< 0.001, η^2= 0.713
Hamstrings intensity (mA)
J)
$F_{(5, 25)}$= 13.28, P< 0.001, η^2= 0.727
Legs intensity (mA)
a
1Hz
2Hz
4Hz
6Hz
8Hz
10Hz
0
20
40
60
80
100
120
140
Frequencies

Figure S3. Impulse intensity at rest (n=6) applying whole-body electromyostimulation with different frequencies. Impulse intensity ranges from 0 mA to 125 mA, being 0 mA "no intensity", and 125 mA "all intensity possible”. *p*-values from repeated measures analysis of variance (ANOVA). Similar letters represent differences between experimental conditions as determined by post-hoc Bonferroni analysis. Data are presented as mean and standard error of the mean (SEM).

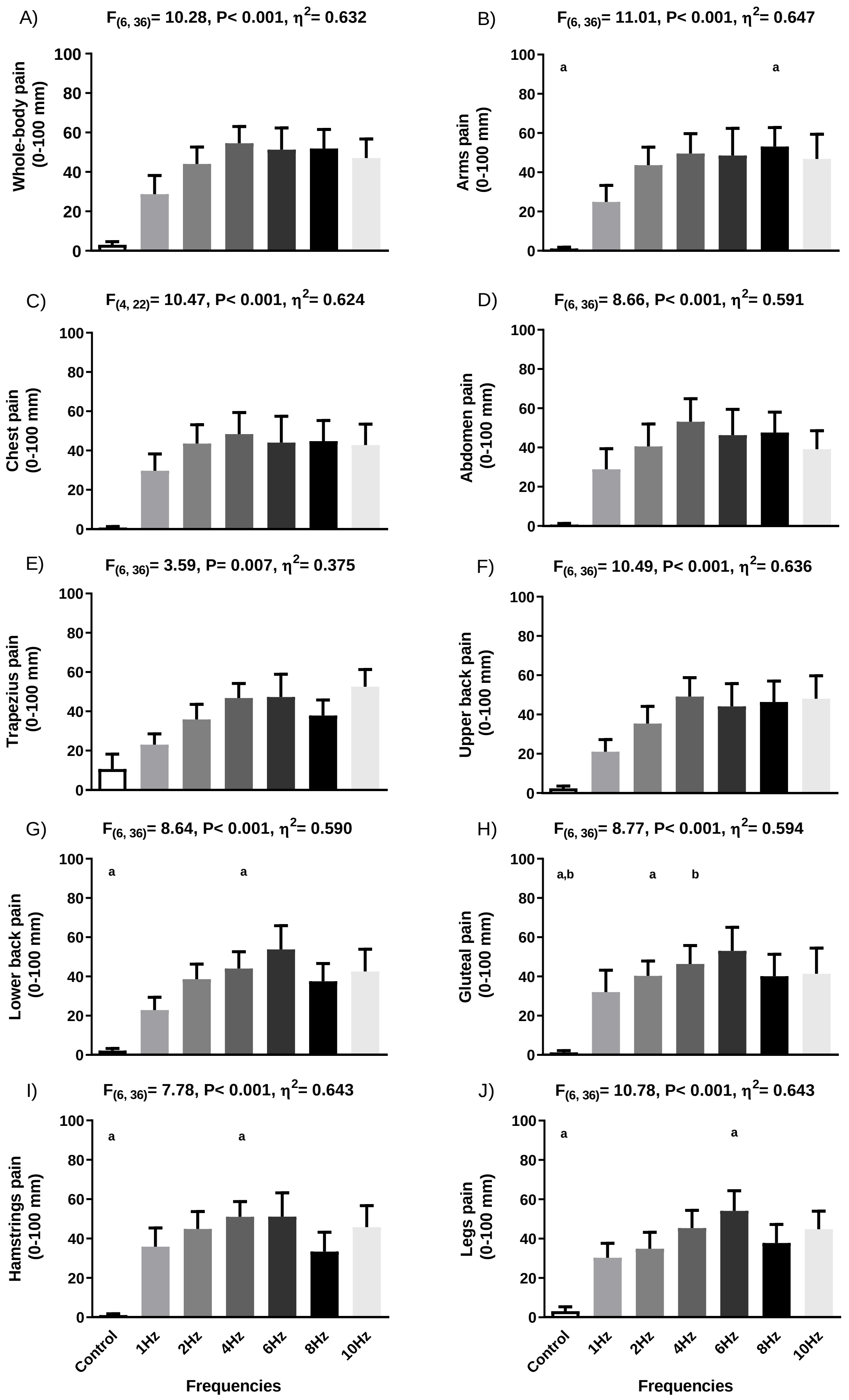

A)
F(6, 36)= 10.28, P< 0.001, η2= 0.632
Whole-body pain (0-100 mm)
B)
F(6, 36)= 11.01, P< 0.001, η2= 0.647
Arms pain (0-100 mm)
C)
F(4, 22)= 10.47, P< 0.001, η2= 0.624
Chest pain (0-100 mm)
D)
F(6, 36)= 8.66, P< 0.001, η2= 0.591
Abdomen pain (0-100 mm)
E)
F(6, 36)= 3.59, P= 0.007, η2= 0.375
Trapezius pain (0-100 mm)
F)
F(6, 36)= 10.49, P< 0.001, η2= 0.636
Upper back pain (0-100 mm)
G)
F(6, 36)= 8.64, P< 0.001, η2= 0.590
Lower back pain (0-100 mm)
H)
F(6, 36)= 8.77, P< 0.001, η2= 0.594
Gluteal pain (0-100 mm)
I)
F(6, 36)= 7.78, P< 0.001, η2= 0.643
Hamstrings pain (0-100 mm)
J)
F(6, 36)= 10.78, P< 0.001, η2= 0.643
Legs pain (0-100 mm)
Control
1Hz
2Hz
4Hz
6Hz
8Hz
10Hz
Frequencies

Figure S4. Pain perception in different anatomic locations after applying whole-body electromyostimulation at different frequencies during uphill walking (n=7). Visual analogue scales (VAS) ranges from 0 to 100, being 0 "no pain", and 100 "the maximum tolerable pain”. *p*-values from repeated measures analysis of variance (ANOVA). Similar letters represent differences between experimental conditions as determined by post-hoc Bonferroni analysis. Data are presented as mean and standard error of the mean (SEM).

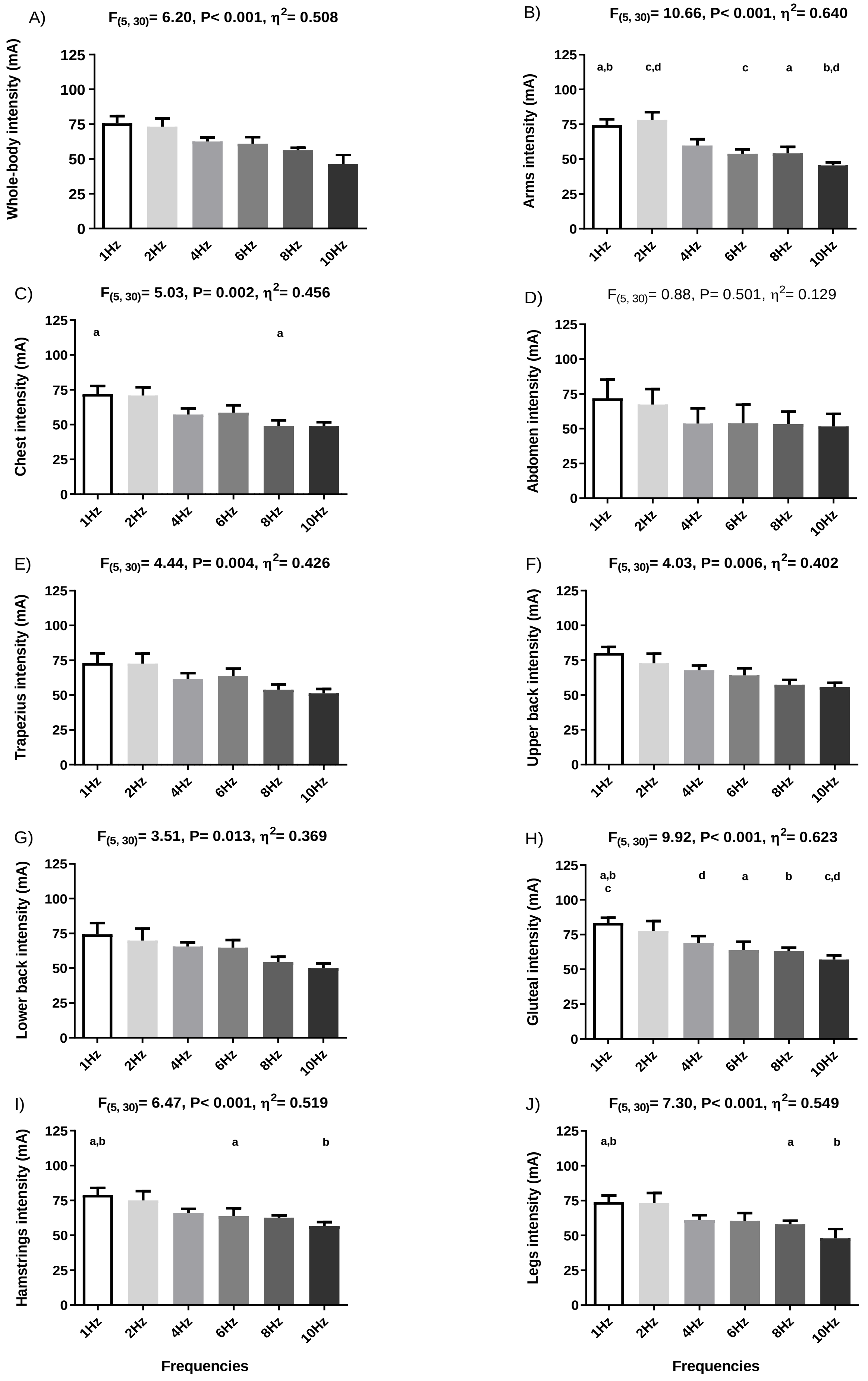
A)
$F_{(5, 30)}$= 6.20, P< 0.001, η^2= 0.508
Whole-body intensity (mA)
B)
$F_{(5, 30)}$= 10.66, P< 0.001, η^2= 0.640
Arms intensity (mA)
a,b
c,d
c
a
b,d
C)
$F_{(5, 30)}$= 5.03, P= 0.002, η^2= 0.456
Chest intensity (mA)
a
a
D)
$F_{(5, 30)}$= 0.88, P= 0.501, η^2= 0.129
Abdomen intensity (mA)
E)
$F_{(5, 30)}$= 4.44, P= 0.004, η^2= 0.426
Trapezius intensity (mA)
F)
$F_{(5, 30)}$= 4.03, P= 0.006, η^2= 0.402
Upper back intensity (mA)
G)
$F_{(5, 30)}$= 3.51, P= 0.013, η^2= 0.369
Lower back intensity (mA)
H)
$F_{(5, 30)}$= 9.92, P< 0.001, η^2= 0.623
Gluteal intensity (mA)
a,b
c
d
a
b
c,d
I)
$F_{(5, 30)}$= 6.47, P< 0.001, η^2= 0.519
Hamstrings intensity (mA)
a,b
a
b
J)
$F_{(5, 30)}$= 7.30, P< 0.001, η^2= 0.549
Legs intensity (mA)
a,b
a
b
0
25
50
75
100
125
1Hz
2Hz
4Hz
6Hz
8Hz
10Hz
Frequencies
Frequencies

Figure S5. Impulse intensity during uphill walking (n=7) applying whole-body electromyostimulation with different frequencies. Impulse intensity ranges from 0 mA to 125 mA, being 0 mA "no intensity", and 125 mA "all intensity possible”. *p*-values from repeated measures analysis of variance (ANOVA). Similar letters represent differences between experimental conditions as determined by post-hoc Bonferroni analysis. Data are presented as mean and standard error of the mean (SEM).

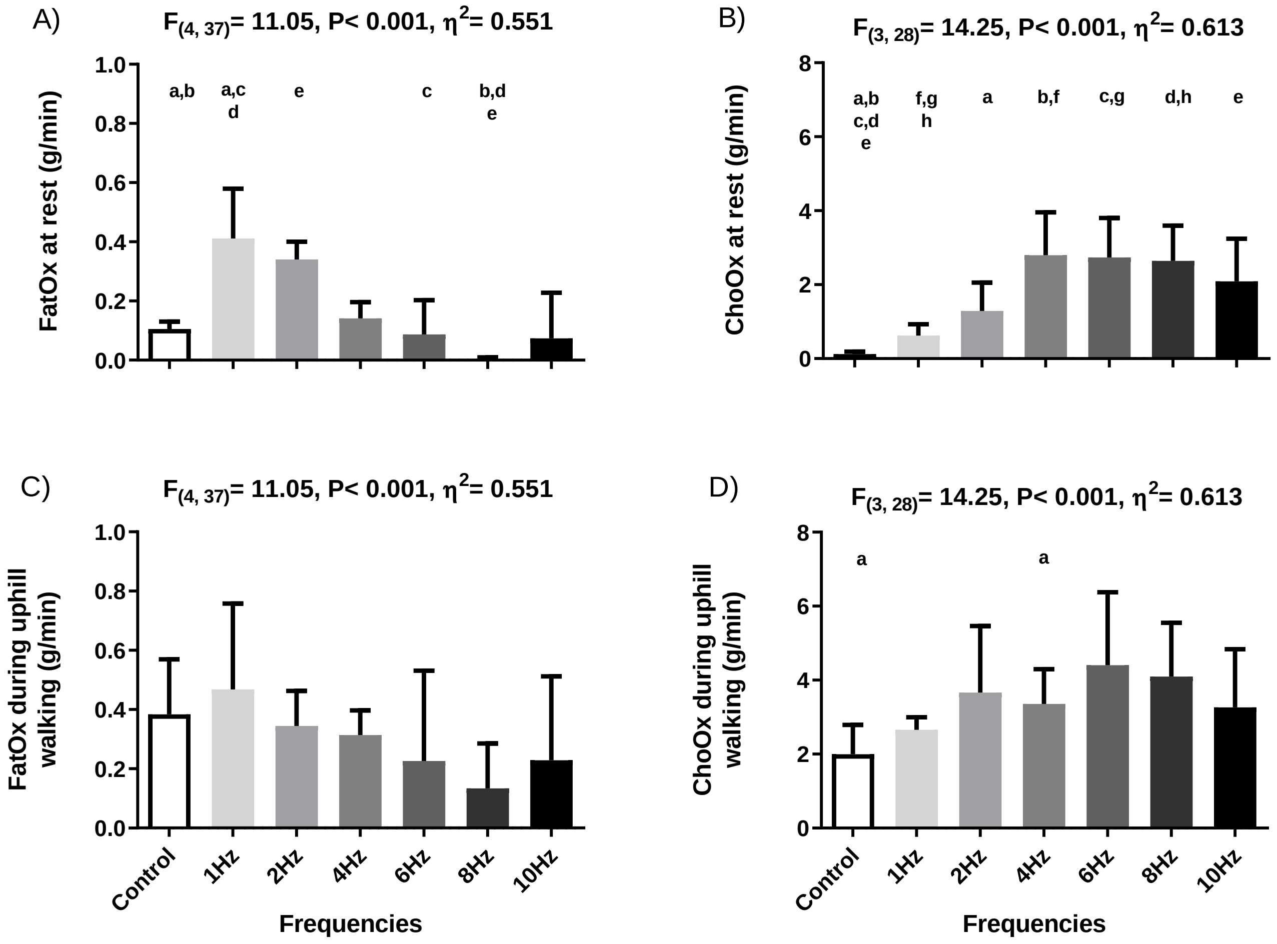

Figure S6. Fat oxidation (FatOx) and carbohydrate oxidation (ChoOx) at rest (n=10) and during uphill walking (n=8) when applying different frequencies of whole-body electromyostimulation. *P*-values from repeated measures analysis of variance (ANOVA). Similar letters represent differences between experimental conditions as determined by post-hoc Bonferroni analysis. Data are presented as mean and standard error of the mean (SEM).
